# Practical and Effective Mentorship Strategies for Caregivers of Children with Chronic Conditions: A Scoping Review

**DOI:** 10.3390/ijerph22030339

**Published:** 2025-02-25

**Authors:** LaDawn N. Duerksen, Chloé Janse van Rensburg, Carrie Costello, Michael A. Golding, Mê-Linh Lê, Maya Woods, Sarah Kelso, Lizabeth Bannister, Jennifer L. P. Protudjer

**Affiliations:** 1 Department of Graduate Studies, University of Manitoba, Winnipeg, MB R3T 2N2, Canada; umletke2@myumanitoba.ca; 2 Department of Food and Human Nutritional Sciences, University of Manitoba, Winnipeg, MB R3T 2N2, Canada; 3 The Children’s Hospital Research Institute of Manitoba, Winnipeg, MB R3E 3P4, Canada; jansevac@myumanitoba.ca (C.J.v.R.); michael.golding@umanitoba.ca (M.A.G.); 4 Department of Pediatrics and Child Health, University of Manitoba, Winnipeg, MB R3A 1S1, Canada; 5 Max Rady College of Medicine, University of Manitoba, Winnipeg, MB R3E 0W2, Canada; 6 Rehabilitation Centre for Children, Winnipeg, MB R3E 3G1, Canadamwoods@rccinc.ca (M.W.);; 7 The Center for Implementation, Winnipeg, MB K0K 1C0, Canada; 8 Neil John Maclean Health Sciences Library, University of Manitoba, Winnipeg, MB R3E 3P5, Canada; me-linh.le@umanitoba.ca; 9 Institute of Environmental Medicine, Karolinska Institutet, 17177 Stockholm, Sweden

**Keywords:** mentors, self-help groups, chronic disease, caregiver burden, caregiver, peer group

## Abstract

Caregivers of children with chronic conditions face daily challenges and a lower quality of life, which may be improved through peer support. This scoping review explored the literature on formal caregiver-to-caregiver mentorship programs, identifying strategies to inform future programs. Using Arskey and O’Malley’s framework, we searched five databases for peer-reviewed literature on caregiver-to-caregiver mentorship programs for adult caregivers caring for children (≤18 years) with chronic conditions. Thematic analysis was performed on relevant articles. Of the 10 064 search hits, 109 were included after full-text screening. Theme 1, “Mentorship adds to medical support”, reflected how mentorship can complement medical care provided by healthcare teams. Theme 2, “Successful mentorship requires the right mentors”, highlighted the qualities of mentors crucial for effective mentorship, mentor-matching practices, and training areas for mentors. Theme 3, “Mentorship programs should balance structure and flexibility”, emphasized the importance of allowing for flexibility to accommodate diverse family needs. Theme 4, “Mentorship programs face common challenges”, summarized the challenges frequently faced when implementing mentorship programs. The study findings suggest that the success of mentorship programs hinge on factors including a flexible program structure, knowledgeable and dedicated mentors, and an infrastructure in place for supporting both the mentors and the financial needs of the program.

## 1. Introduction

Caregivers of children living with a chronic condition may face challenges beyond those common to raising a child without a chronic condition. Challenges include an increased financial burden, the lack of acceptance and support from others, and the responsibility of caring for a child with intense needs [1]. These additional challenges increase their risk of psychosocial burdens and may lower their quality of life [2,3].

Supportive social interactions with family members, friends, and one’s community can reduce stress and improve the resiliency for caregivers of children with a chronic condition [1]. The Centers for Disease Control and Prevention defines chronic diseases as ‘conditions that last 1 year or more and require ongoing medical attention or limit activities of daily living or both’ [4]. Examples in childhood include, but are not limited to, allergies, asthma, blindness, cystic fibrosis, deafness, diabetes, and neurodevelopmental disorders (NDDs), such as delayed intellectual development, autism spectrum disorder, and attention deficit hyperactivity disorder (ADHD) [5]. Peer support from other caregivers of children with similar chronic conditions provides another level of support. Fellow caregivers may understand what life with a chronically ill child is like and can offer practical advice based on lived experience [6].

According to Ragins and Kram [7], mentoring can be defined as “a relationship between an older, more experienced mentor and a younger, less experienced protégé for the purpose of helping and developing the protégé’s career”. While early literature about mentorship in the 1980s focused on career development, such as the work performed by Kram and Isabella [8], the idea of mentorship as a whole has continued to evolve with time. Many different models for mentorship exist today—for example, according to Clutterbuck [9], developmental mentorship is “a helping relationship based on an exchange of knowledge, experience and goodwill”. Within the context of caregivers of children with chronic conditions, the idea of mentorship can be applied in a broader manner similar to Clutterbuck’s description—focusing on emotional and informational support rather than career advancement.

In general, caregiver-to-caregiver mentorship can be formal or informal. Informal mentorships may be provided by family, friends, or other connections and are not organizationally regulated [10]. Many of these informal mentor–mentee relationships form spontaneously. For example, a caregiver of a child with a newly diagnosed chronic condition is introduced to another caregiver, who also has a child with a chronic condition, through a mutual friend. Formal mentorship, on the other hand, is regulated by an organization [10], often in the form of a mentorship program. Appropriate matches between mentors and mentees are typically determined internally, and the program may have a more rigid set of documented expectations and guidelines [11,12].

While evidence for the role of peer support on alleviating caregiver burden is abundant, little research has been performed on developing caregiver-to-caregiver mentorship programs or the factors that make them successful. Herein, we aimed to fill this gap by performing a scoping review of the literature on formal mentorship for caregivers of children with chronic conditions in order to identify the factors that either contribute or detract from the program’s success. We hope that by consolidating and synthesizing this information, future researchers, clinicians, and program administrators will be better equipped to not only develop successful mentorship programs, but also improve and maintain existing ones.

## 2. Materials and Methods

Our methods broadly followed the methodological frameworks outlined by Arksey and O’Malley, and Levac et al. [13,14]. Our scoping review is in compliance with the PRISMA guidelines.

### 2.1. Eligibility Criteria

Articles were considered eligible if they were original articles that referred to the formal peer mentorship of an adult caregiver caring for a child, aged 0–18 years, with a chronic condition. For the purposes of this review, our definition of peer support was loosely drawn from the description by Dodds and Singer [15]. Peer support was defined as any formal, or planned, connection between caregivers of children with chronic conditions (as defined by the CDC above) that provided affirmational, emotional, or informational support [4,15]. Articles were limited to English and French language publications only, but no limitations on date were applied. Articles about peer mentorship programs for caregivers of children without a chronic condition or children over 18 years were excluded. Review articles were also excluded.

### 2.2. Databases and Search Strategy

The search strategy was developed by a health sciences librarian M.L. in consultation with the research team as well as the Rehabilitation Centre for Children Family Advisory Council. The search, which used a combination of subject headings and keywords was developed in Ovid MEDLINE and peer-reviewed by a second librarian according to the Peer Review of Electronic Search Strategies (PRESS) 2015 Guideline Statement. The search was then translated with appropriate subject headings and keywords for the other databases. No date limits were applied but the results were limited to the English and French languages only. The search was run on 4 May 2023 in MEDLINE (Ovid), PsycInfo (Ovid), Cochrane Library (Wiley), CINAHL (EBSCOhost), and Scopus. The results were uploaded into Covidence (Veritas Health Innovation. Melbourne, Australia) for deduplication. All of the search strategies and results are available at: https://doi.org/10.5683/SP3/TVCC3V and the full search strategy for MEDLINE (Ovid) can be found in Table S1.

Additionally, gray literature sites were searched from September to November 2023 by using the following terms: mentor*, caregiv*, support*, parent*, partner*, and navigat*. English- and French-language publications only were scanned, and the searches were limited to the first 10 pages of hits from each database. There were no limits on the dates of publications.

### 2.3. Selection of Studies

The screening process began by creating a Covidence test project with 50 random references. The references were then screened by two separate researchers to test the inter-rater reliability. Afterwards, the project’s writing team L.D. and C.J. virtually to discuss the test and clarify the inclusion criteria. The proportional agreement between the two raters was 82%.

Once the inclusion criteria were clear, titles and abstracts for the remaining articles were independently screened for relevance by the same researchers L.D. and C.J. and designated as ‘yes’, ‘no’, or ‘maybe’ in the Covidence database. Duplicate articles, as well as articles that were not deemed to be relevant to the research question, were removed, with conflicts between the researchers resolved through virtual discussion. A third author J.P. was available to settle any conflicts that could not be resolved through discussion.

### 2.4. Data Charting

An Excel data charting spreadsheet was developed to extract data from the selected articles and publications. The form included fields for the last name of the first author, title, year of publication, country/countries where the participants were recruited from, the aim of the article or publication, the sample type and size, the study type, the recruitment strategy, the method of evaluation, the key findings, the program purpose, the program length, the program components, the training method, the program modality, the topics covered, and the recommendations given. Once data extraction was completed, two researchers double-checked the entries by randomly selecting ten articles to assess for accuracy.

### 2.5. Thematic Analysis

The descriptive characteristics from the extracted data were reviewed independently by two research assistants to identify qualitative themes in the literature through a thematic analysis approach. As part of this approach, extracted data from each of the studies were organized by applying descriptive codes, known as subthemes, to relevant sections of the text. Once coding was completed, the research assistants independently grouped subthemes that were conceptually similar into broader themes. These themes captured a central concept conveyed by the collection of subthemes related to the research question rather than describing one idea. The research assistants then met to discuss individual findings, and four main themes were agreed upon.

As a scoping review of previously published literature, no research ethics board approval was required or sought.

## 3. Results

### 3.1. Study Selection

**Figure 1:**
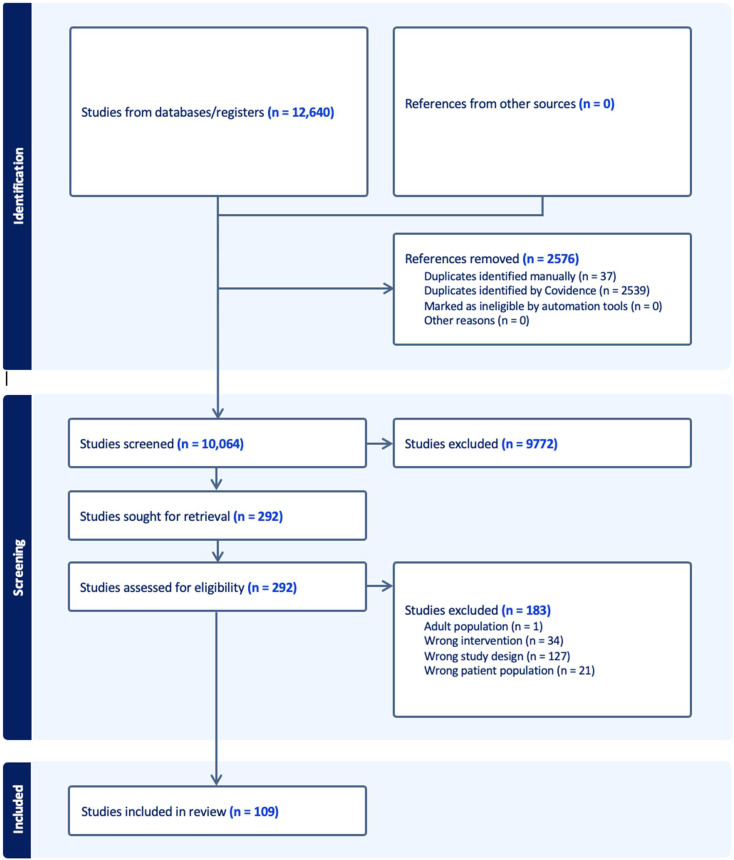
Flow diagram of the studies included.

In total, 12,640 articles were retrieved from the database search. A total of 2539 duplicate articles were removed by Covidence and 37 were removed manually, resulting in 10,064 articles. After title and abstract screening, 292 articles were selected for full-text screening. A total of 109 (1.08%) of the articles met the final inclusion criteria and were included in the scoping review (Figure 1). The gray literature search yielded no relevant articles. 

All of the included articles were published between 1978 and 2022, and the majority were collectively conducted in the United States (*n* = 67). Other articles were conducted in Canada (*n* = 14), both the United States and Canada (*n* = 2), the United Kingdom (*n* = 15), Australia (*n* = 5), or other countries (*n* = 6). A total of 36 of the articles were qualitative, 18 were quantitative, 49 were mixed-methods, and 8 were categorized as ‘other’, including program descriptions and theoretical papers. A table with all of the extracted data from the included studies can be found in Table S2.

### 3.2. Thematic Results

**Figure 2:**
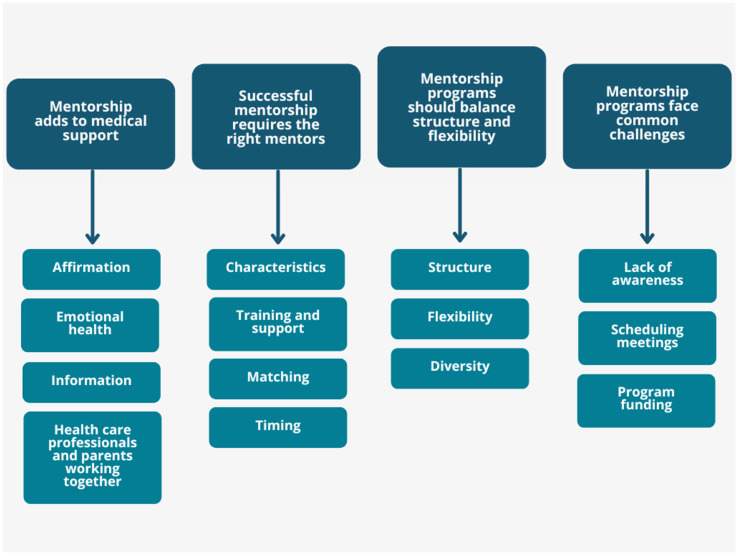
Summary of themes.

Based on the extracted data from the included articles, several subthemes were identified. From these subthemes, four major themes were established, related to formally organized mentorship programs for caregivers of children with chronic conditions, as follows: (1) Mentorship adds to medical support; (2) Successful mentorship requires the right mentors; (3) Mentorship programs should have a balance of structure and flexibility; (4) Mentorship programs face common challenges. Four subthemes were grouped to form theme 1, including affirmational support, improved emotional health, practical information, and healthcare professionals and parents working together. The four subthemes for theme 2 were the characteristics of the mentors, training and support, matching, and timing. The three subthemes used for theme 3 were structure, flexibility, and diversity. Finally, the following three subthemes were grouped to form theme 4: lack of awareness about programs, difficulty scheduling meetings, and lack of program funding. These themes and subthemes are summarized in Figure 2.

#### 3.2.1. Theme 1: Mentorship Adds to Medical Support

A key finding from the reviewed literature is the complementary nature of caregiver-to-caregiver mentorship to traditional medical support [16,17,18,19,20,21,22]. While medical interventions address the physical needs of care recipients, mentorship programs offer invaluable psychosocial benefits through affirmational, emotional, and informational support [20,23,24,25,26,27,28,29,30,31,32,33,34,35,36,37]. Affirmational support [28,29,37,38,39] from a mentor was found to increase a mentee’s acceptance of their child’s condition, their confidence in their ability to adapt, and their perspective about the future [19,34,40,41,42,43,44,45,46]. Mentees often reported that they developed better coping strategies and felt more hopeful as a result of this affirmational support. Mentors also provide unique emotional support by creating a sense of belonging and connection through their shared experiences [6,22,32,42,43,47,48,49,50,51,52,53,54]. The sense of belonging and connection originating from shared experiences was reported to reduce stress, anxiety, and depression [18,29,44,55,56,57,58,59,60,61,62,63,64]. Informational support, in the way of practical day-to-day navigational support, fosters a sense of empowerment among caregivers, enabling them to make better informed decisions about their caregiving roles and responsibilities. For example, mentors are able to share tips drawn from their personal lived experiences with their child or those that they learnt through their training for the program [23,37,44,55,57,65,66,67,68,69,70]. These tips may be related to the skills needed to manage their child’s condition [32,34,37,55,57,65,66,69,71,72,73,74] or navigation of school and the healthcare system more broadly [18,23,57,58,67,68,72,75,76,77,78,79]. Mentors also have the ability to share relevant community supports with caregivers, whether these resources were discovered through their own experiences or provided to them during the program’s training [19,23,24,25,26,27,31,36,37,43,49,52,57,65,72,80,81,82,83,84,85]. In these ways, caregiver-to-caregiver mentorship acts as a liaison between medical healthcare teams and caregivers, enhancing medical support and supporting caregivers by making health information more accessible and relatable.

#### 3.2.2. Theme 2: A Successful Mentorship Program Requires the Right Mentors

Central to the effectiveness of mentorship programs is the selection of appropriate mentors who possess the necessary qualities and experiences to provide meaningful support. Mentors should demonstrate empathy, positivity, resilience, good communication skills, and a genuine commitment to supporting their mentees [15,35,37,65,70,86]. Additionally, mentors should receive training in a variety of areas to ensure they have the skills needed to provide empathetic and informed support. This training may be related to listening skills [6,21,24,29,46,52,66,67,77,80,82,83,84,87,88,89,90,91], sharing stories [29,59,77,83,88,89,92], handling strong emotions [43,59,93], empowerment [23,41,43,72,80], and boundary setting [23,24,56,72,77,80,84,88,92,93,94]. Mentors may also receive more experiential and hands-on training through modalities such as role playing [6,23,24,26,29,30,57,80,82,88,90,95,96,97] and shadowing [23,71,91]. All of the above areas of training can be administered in several ways, such as in-person sessions, reading materials or modules, and workshops. Training sessions may also vary in length from a few hours, to single-day sessions, to multiple sessions over several weeks or months. Beyond training, the success of a mentorship program hinges on a successful relational match between a mentor and mentee based on a key sameness factor, such as culture, geographical location, child’s health issue, stage of life, or other similar characteristics [15,16,19,24,33,45,51,52,53,61,75,85,92,98,99]. Furthermore, the timing of mentorship interactions is crucial in terms of mentorship initiation and maintenance. Ideally, potential mentees should be introduced to the program soon after receiving a diagnosis for their child, which may be facilitated by their clinician [27,30,41,67,78]. Additionally, mentors should be available when caregivers are most in need of guidance and encouragement [15,27,30,41,67,100].

#### 3.2.3. Theme 3: Mentorship Programs Should Balance Structure and Flexibility

Successful caregiver-to-caregiver mentorship programs have a clear mission and structure while allowing for flexibility to meet the diverse needs of families. Important structural components include a planning committee to oversee the development of the program [34,95], written policies and guidelines [19,47,78,97], documentation of mentor–mentee connections [33,80,86,101], feedback surveys [100,102], and a paid coordinator to manage logistics [19,20,28,33,53,65,67,68,71,78,83,89,91,98]. Some caregivers may prefer structured mentorship sessions with predefined topics and goals, and others may prefer more informal interactions tailored to their specific concerns [6,17,24,26,31,46,51,53,55,68,75,78,84,89,95,98,101,103,104]. By offering a variety of mentorship formats, such as one-on-one meetings, support groups, and online forums, programs can accommodate the preferences and schedules of caregivers, enhancing engagement and retention.

#### 3.2.4. Theme 4: Mentorship Programs Face Common Challenges

Mentorship programs encounter common challenges that must be addressed to maximize their impact. Common challenges include recruiting and retaining qualified mentors [30,105], preventing mentor burnout [62,95,102], matching mentors with compatible mentees [16,51], ensuring ongoing support and supervision for mentors [47,58], and scheduling meetings to accommodate busy schedules, childcare, and transportation needs [16,30,51,70,98,100,105,106]. Additionally, program funding can be a substantial hurdle, thus highlighting the need for long-term commitment and strategic partnerships with interested parties [16,81,92,107,108].

## 4. Discussion

This scoping review aimed to explore and summarize the current literature related to formal mentorship for caregivers of children with chronic conditions. Through the thematic analysis of 109 included articles, four consistent themes were identified related to such mentorship programs. The theme, Mentorship adds to medical support, highlights the finding that caregiver-to-caregiver mentorship compliments the medical support provided by healthcare providers. A successful mentorship program requires the right mentors outlines the qualities that are beneficial for mentors to have, and emphasizes the importance of proper training and mentor-matching practices. Mentorship programs should balance structure and flexibility describes the benefits of having a paid program coordinator and the importance of meeting the diverse needs of families by accommodating various accessibility needs or preferences. Finally, Mentorship programs face common challenges summarizes the difficulties reported when implementing and managing mentorship programs. Overall, these themes reflect the benefits and challenges related to mentorship programs, as well as factors to keep in mind to ensure effective and efficient caregiver-to-caregiver mentorship. These themes can further be used to aid in the development, implementation, and management of current or future mentorship programs.

The findings that make up the theme Mentorship adds to medical support emphasize that caregiver-to-caregiver mentorship is a valuable resource for families in a variety of capacities—from aiding caregivers in making informed decisions [6,57,64,69,74,103] to reducing depression and anxiety [19,29,56,60]. These findings are consistent with a systematic review by Wong and Shorey [109], who found that caregivers of children with NDDs typically sought out peer support because they felt isolated and unsure of how to find the support their child needed. Peer support was found to improve mental well-being and quality of life, and thus Wong and Shorey [109] recommended peer support as an adjunct to healthcare services. It is important to note that Wong and Shorey’s paper had a focus on qualitative studies related to peer support for caregivers of children with NDDs, whereas this review has a broader approach. With regards to program development, many programs reported the use of a multidisciplinary team during development, drawing expertise from parents as well as medical staff [16,17,22,83,93,102]. This further aids in the ability of mentorship programs to cater to their caregiver population while bridging the gap with healthcare teams.

Through the theme A successful mentorship program requires the right mentors, our scoping review highlights that interpersonal dynamics between mentors and mentees are a determining factor for the success of any program, and careful mentor-to-mentee matching must be a priority. A strong match can help ensure caregivers feel that they are in a safe and supportive environment, where they are able to openly discuss their concerns. These findings were also consistent with the findings of Wong and Shorey [109], who found that creating a bond through shared experiences was extremely important to families. Matching can be based on various factors and how to do this successfully is an area for future research.

As seen from the theme Mentorship programs should balance structure and flexibility, the literature on caregiver mentorship highlights the core administrative factors that contribute to an effective mentorship program, most specifically the importance of having a program coordinator. Program coordinators have a wide variety of roles within the scope of mentorship programs—they are able to help conduct the training of mentors, arrange matches, and have check-in meetings with mentors and caregivers [19,28,33,42,65,67,78,88,89,100,110]. The importance of having a program coordinator is also highlighted by Wong and Shorey [109], who found that caregivers felt peer programs lacked the coordinators needed to provide clarity through management and oversight.

Mentorship programs face common challenges, our fourth and final theme, outlines the frequently mentioned barriers faced when creating and implementing programs. The challenges mentioned include a lack of funding, difficulties with matching due to incompatibility, and logistical concerns such as scheduling issues or travel costs. Wong and Shorey [109] also identified several of these barriers in their review, stating that difficulties included “geographical distances, conflicting commitments, lack of time, and other work related stressors” [109]. It is important to note that these barriers apply to both the caregivers and the mentors themselves. These common challenges cited in the literature underscore the gaps that must be addressed when developing future mentorship programs in order to ensure that the program is sustainable and provides benefits to all.

By examining the specific, recurring findings that arise across the literature, we are able to ensure that important recommendations can be taken into consideration for the development and implementation of new mentorship programs. Broadly examining and grouping ideas from the literature additionally facilitates a more comprehensive perspective of caregiver-to-caregiver mentorship.

### 4.1. Strengths and Limitations

Our scoping review search method resulted in the retrieval of a large dataset and resulted in a comprehensive understanding of mentorship programs as a whole. The consistency of the findings throughout the included studies lends some confidence to the results of our study, and our large sample size helps to ensure the findings generalize, to some degree, across chronic conditions, including NDDs.

Only articles published in English or French were included in our study, and the search terms used were in English, possibly excluding other research that has been conducted on this topic. Additionally, the demographics of our included studies were not specifically analyzed or compared; though, with such a large volume of data, it can be speculated that a variety of populations were recruited, making our results generalizable.

Finally, the heterogeneity of the included studies and the lack of standardized methods to assess the success of mentorship programs represent notable limitations. Such variations can make it challenging to draw definitive conclusions or to compare results directly, as different studies might use various definitions, metrics, and protocols to assess similar concepts. This represents a gap in the literature and makes it difficult to definitively recommend best practices or to implement evidence-based policies. Therefore, future research should aim to develop standardized definitions and practice guidelines for caregiver mentorship programs, screening tools for mentor selection, matching protocols, and common program evaluation strategies.

### 4.2. Implications for Future Research and Practice

Healthcare policy makers should consider the findings of this study and consider integrating caregiver mentorship programs into support systems. Sufficient financial resources should be provided to ensure the quality and sustainability of such programs. The benefits described in this study are likely to reduce future healthcare costs. For example, caregivers who are supported by mentors may make better informed decisions for their children, which, in turn, may reduce the number of medical appointments needed. This provides an incentive for healthcare centers to provide this upstream funding. Koch and Jones [111] report that support for caregivers has a direct positive impact on the well-being of their children, which is yet another incentive for mentorship programming.

Mentorship program development needs to take into consideration the barriers found in the literature and proactively make efforts to prevent burnout for mentors. Recruiting efforts should be focused on identifying individuals who already have strong self-reflexive and self-care strategies [111]. This can be achieved by partnering with organizations that already support children with chronic conditions, as well as reaching out to healthcare professionals, social workers, and community leaders who have first-hand knowledge of caregivers with these traits. Providing thorough and on-going training for mentors and caregivers is essential. This training should include mentorship, and relational and self-care skills [111]. Additionally, offering continuous support and resources to mentors helps in retaining them. This support can come in the form of regular check-ins, access to a support network of other caregivers, and resources for dealing with the challenges they face. Future research should also further investigate the effects of mentor burnout, and investigate strategies to address and prevent it.

## 5. Conclusions

The findings from this scoping review underscore the significant role that caregiver-to-caregiver mentorship programs play in complementing traditional medical support for families of children with chronic conditions, offering critical psychosocial benefits that enhance the overall well-being of caregivers. Based on this study’s findings, healthcare providers are urged to consider integrating mentorship programs within their support services. In addition, the importance of the quality training of mentors and thorough mentor-matching practices cannot be understated in the successful implementation of these programs. Future programs should also consider hiring a program coordinator to facilitate clear communication between mentors and caregivers, ensuring the program runs smoothly. Overall, these findings emphasize areas that should be prioritized during the creation and implementation of caregiver-to-caregiver mentorship programs.

## Data Availability

The datasets generated and/or analyzed during the current study are available in the Borealis repository, https://doi.org/10.5683/SP3/TVCC3V.

## Supplementary Materials

The following supporting information can be downloaded at: https://www.mdpi.com/article/10.3390/ijerph22030339/s1, Table S1: MEDLINE (Ovid) Full search string used to identify available peer-reviewed literature; Table S2: Extracted data from included studies.

## Abbreviations

The following abbreviations are used in this manuscript:

ADHD	attention deficit hyperactivity disorder
NDDs	neurodevelopmental disorders

## References

[1] Faden S.Y., Merdad N., Faden Y.A. (2023). Parents of Children With Neurodevelopmental Disorders: A Mixed Methods Approach to Understanding Quality of Life, Stress, and Perceived Social Support. Cureus.

[2] Reis G.A., Zonta J.B., Camilo B.H.N., Fumincelli L., Gonçalves A.M.D.S., Okido A.C.C. (2020). Qualidade de Vida de Cuidadores de Crianças Com Transtornos Do Neurodesenvolvimento. Rev. Eletr. Enferm..

[3] Turnage D., Conner N. (2022). Quality of Life of Parents of Children with Autism Spectrum Disorder: An Integrative Literature Review. Spec. Pediatr. Nurs..

[4] National Centre for Chronic Disease Prevention and Health Promotion About Chronic Diseases. https://www.cdc.gov/chronicdisease/index.html.

[5] World Health Organization Mental Disorders Fact Sheet. https://www.who.int/news-room/fact-sheets/detail/mental-disorders.

[6] Moody E.J., Kaiser K., Sharp D., Kubicek L.F., Rigles B., Davis J., McSwegin S., D’Abreu L.C., Rosenberg C.R. (2019). Improving Family Functioning Following Diagnosis of ASD: A Randomized Trial of a Parent Mentorship Program. J. Child Fam. Stud..

[7] Eleanor R., Justice Sandra D.O., Ragins B.R., Kram K.E. (2008). The Roots and Meaning of Mentoring. The Handbook of Mentoring at Work: Theory, Research, and Practice.

[8] Kram K.E., Isabella L.A. (1985). Mentoring alternatives: The role of peer relationships in career development. Acad. Manag. J..

[9] Clutterbuck D. (2013). Making the Most of Developmental Mentoring: A Practical Guide for Mentors and Mentees.

[10] Chao G.T., Walz P., Gardner P.D. (1992). Formal and informal mentorships: A comparison on mentoring functions and contrast with nonmentored counterparts. Pers. Psychol..

[11] Germeroth D., Murray C.M., McMullen-Roach S., Boshoff K. (2024). A Scoping Review of Mentorship in Allied Health: Attributes, Programs and Outcomes. Aust. Occup. Ther. J..

[12] Goh S., Wong R.S.M., Quah E.L.Y., Chua K.Z.Y., Lim W.Q., Ng A.D.R., Tan X.H., Kow C.S., Teo Y.H., Lim E.G. (2022). Mentoring in Palliative Medicine in the Time of Covid-19: A Systematic Scoping Review. BMC Med. Educ..

[13] Levac D., Colquhoun H., O’brien K.K. (2010). Scoping Studies: Advancing the Methodology. Implement. Sci..

[14] Arksey H., O’Malley L. (2005). Scoping Studies: Towards a Methodological Framework. Int. J. Soc. Res. Methodol. Theory Pract..

[15] Dodds R.L., Singer G.H.S. (2018). Parent-to-Parent Support Providers: How Recruits Are Identified. J. Appl. Res. Intellect. Disabil..

[16] Ainbinder J.G., Blanchard L.W., Singer G.H.S., Ellen Sullivan M., Powers L.K., Marquis J.G., Santelli B. (1998). A Qualitative Study of Parent to Parent Support for Parents of Children With Special Needs MEd, and the Consortium to Evaluate Parent to Parent. J. Pediatr. Psychol..

[17] Cherniss D.S. (1987). Stability and Growth in Parent-Support Services: A National Survey of Peer Support for Parents of Premature and High-Risk Infants. Research on Support for Parents and Infants in the Postnatal Period.

[18] Hopkins L., Kuklych J., Pedwell G., Woods A. (2021). Supporting the Support Network: The Value of Family Peer Work in Youth Mental Health Care. Community Ment. Health J..

[19] Levick J., Quinn M., Vennema C. (2014). NICU Parent-to-Parent Partnerships: A Comprehensive Approach. Neonatal Netw..

[20] Mehta K., Hilton E., Baldwin M., Watkin P. (2020). Parent-to-Parent Support for the Families of Deaf Children Identified by the Newborn Hearing Screen. Deaf. Educ. Int..

[21] Pollock M.D., Ming D., Chung R.J., Maslow G. (2022). Parent-to-Parent Peer Support for Children and Youth with Special Health Care Needs: Preliminary Evaluation of a Family Partner Program in a Healthcare System. J. Pediatr. Nurs..

[22] Solomon M., Pistrang N., Barker C. (2001). The Benefits of Mutual Support Groups for Parents of Children With Disabilities. Am. J. Community Psychol..

[23] Carty C.L., Soghier L.M., Kritikos K.I., Tuchman L.K., Jiggetts M., Glass P., Streisand R., Fratantoni K.R. (2018). The Giving Parents Support Study: A Randomized Clinical Trial of a Parent Navigator Intervention to Improve Outcomes after Neonatal Intensive Care Unit Discharge. Contemp. Clin. Trials.

[24] Channon S., Lowes L., Gregory J.W., Grey L., Sullivan-Bolyai S. (2016). Feasibility of Parent-to-Parent Support in Recently Diagnosed Childhood Diabetes: The PLUS Study. Diabetes Educ..

[25] Krantz C., Hynes M., DesLauriers A., Kitcher L., MacMillan T., Paradis D., Curry S. (2021). Helping Families Thrive: Co-Designing a Program to Support Parents of Children with Medical Complexity. World Health Popul..

[26] Duppong Hurley K., Huscroft-D’Angelo J. (2018). Parent Connectors: A Parent-to-Parent Support Program Feasible for Rural Settings. Rural. Spec. Educ. Q..

[27] Hartman A.F., Radin M.B., Mcconnell B. (1992). Parent-to-Parent Support: A Critical Component of Health Care Services for Families. Issues Compr. Pediatr. Nurs..

[28] Ireys H.T., Sills E.M., Kolodner K.B., Walsh B.B. (1996). A Social Support Intervention for Parents of Children with Juvenile Rheumatoid Arthritis: Results of a Randomized Trial. J. Pediatr. Psychol..

[29] Ireys H.T., Chernoff R., Devet K.A., Kim Y. (2001). Maternal Outcomes of a Randomized Controlled Trial of a Community-Based Support Program for Families of Children With Chronic Illnesses. Arch. Pediatr. Adolesc. Med..

[30] Iscoe L., Bordelon K. (1985). Pilot Parents: Peer Support for Parents of Handicapped Children. Child. Health Care.

[31] Kremkow J.M.D., Finke E.H. (2022). Peer Experiences of Military Spouses with Children with Autism in a Distance Peer Mentoring Program: A Pilot Study. J. Autism Dev. Disord..

[32] Law M., King S., Stewart D., King G. (2002). The Perceived Effects of Parent-Led Support Groups for Parents of Children with Disabilities. Phys. Occup. Ther. Pediatr..

[33] Lindsay J.K., Roman L., DeWys M., Eager M., Levick J., Quinn M. (1993). Creative Caring in the NICU: Parent-to-Parent Support. Neonatal Netw..

[34] Mann H.G., Sevigny-Skyer S. (2019). Deaf Patients and Children: An Innovative Organization by and for Deaf. J. Am. Deaf. Rehabil. Assoc..

[35] Mills A.S., Vimalakanthan K., Sivapalan S., Shanmugalingam N., Weiss J.A. (2021). Brief Report: Preliminary Outcomes of a Peer Counselling Program for Parents of Children with Autism in the South Asian Community. J. Autism Dev. Disord..

[36] Silver E.J., Ireys H.T., Bauman L.J., Stein R.E.K. (1997). Psychological Outcomes of a Support Intervention in Mothers of Children with Ongoing Health Conditions: The Parent-to-Parent Network. J. Community Psychol..

[37] Sullivan-Bolyai S., Lee M.M. (2011). Parent Mentor Perspectives on Providing Social Support to Empower Parents. Diabetes Educ..

[38] Sullivan-Bolyai S., Grey M., Deatrick J., Gruppuso P., Giraitis P., Tamborlane W. (2004). Helping other mothers effectively work at raising young children with type 1 diabetes. Diabetes Educ..

[39] Evans M., Tang P.Y., Bhushan N., Fisher E.B., Valovcin D.D., Castellano C. (2020). Standardization and Adaptability for Dissemination of Telephone Peer Support for High-Risk Groups: General Evaluation and Lessons Learned. Transl. Behav. Med..

[40] Akre C., Ramelet A.S., Berchtold A., Suris J.C. (2015). Educational Intervention for Parents of Adolescents with Chronic Illness: A Pre-Post Test Pilot Study. Int. J. Adolesc. Med. Health.

[41] Baron Nelson M., Riley K., Arellano K. (2018). Adding a Parent to the Brain Tumor Team: Evaluating a Peer Support Intervention for Parents of Children With Brain Tumors. J. Pediatr. Oncol. Nurs..

[42] Bray L., Carter B., Sanders C., Blake L., Keegan K. (2017). Parent-to-Parent Peer Support for Parents of Children with a Disability: A Mixed Method Study. Patient Educ. Couns..

[43] Butera-Prinzi F., Charles N., Heine K., Rutherford B., Lattin D. (2010). Family-to-Family Link Up Program: A Community-Based Initiative Supporting Families Caring for Someone with an Acquired Brain Injury. NeuroRehabilitation.

[44] Kerr S.M., McIntosh J.B. (2000). Coping When a Child Has a Disability: Exploring the Impact of Parent-to-Parent Support. Child Care Health Dev..

[45] Mangurten H.H., Slade C., Fitzsimons D. (1979). Parent—Parent Support in the Care of High—Risk Newborns. J. Obstet. Gynecol. Neonatal Nurs..

[46] Santelli B., Turnbull A., Higgins C. (1997). Parent to Parent Support and Health Care. Pediatr. Nurs..

[47] Barlow J., Swaby L., Turner A. (2008). Perspectives of Parents and Tutors on a Self-Management Program for Parents/Guardians of Children with Long-Term and Life-Limiting Conditions: “A Life Raft We Can Sail along With”. J. Community Psychol..

[48] Batchelor M., Maguire S., Shearn J. (2021). “They Just Get It” an Exploration of Father’s Experiences and Perceptions of a Support Group for Men Caring for Children with Disabilities and/or Developmental Delay. J. Appl. Res. Intellect. Disabil..

[49] Blake L., Bray L., Carter B. (2019). “It’s a Lifeline”: Generating a Sense of Social Connectedness through Befriending Parents of Disabled Children or Children with Additional Need. Patient Educ. Couns..

[50] McCabe H. (2008). The Importance of Parent-to-Parent Support among Families of Children with Autism in the People’s Republic of China. Int. J. Disabil. Dev. Educ..

[51] Nicholas D.B., Keilty K. (2007). An Evaluation of Dyadic Peer Support for Caregiving Parents of Children with Chronic Lung Disease Requiring Technology Assistance. Soc. Work. Health Care.

[52] Santelli B., Turnbull A.P., Marquis J.G., Lerner E.P. (1995). Parent to Parent Programs: A Unique Form of Mutual Support. Infants Young Child..

[53] Shilling V., Bailey S., Logan S., Morris C. (2015). Peer Support for Parents of Disabled Children Part 2: How Organizational and Process Factors Influenced Shared Experience in a One-to-One Service, a Qualitative Study. Child Care Health Dev..

[54] Ritchie J., Stewart M., Ellerton M.-L., Thompson D., Halifax D.D.H., Scotia N., Meade D., Weld M.A.P. (2000). Parents’ Perceptions of the Impact of a Telephone Support Group Intervention. J. Fam. Nurs..

[55] Dababnah S., Kim I., Magaña S., Zhu Y. (2023). Parents Taking Action Adapted to Parents of Black Autistic Children: Pilot Results. J. Policy Pract. Intellect. Disabil..

[56] Dykens E.M., Fisher M.H., Lounds Taylor J., Lambert W., Miodrag N. (2014). Reducing Distress in Mothers of Children With Autism and Other Disabilities: A Randomized Trial. Pediatrics.

[57] Hock R.M., Rovane A.K., Feinberg M.E., Jones D.E., Holbert A.A. (2022). A Pilot Study of a Co-Parenting Intervention for Parents of Children with Autism Spectrum Disorder. J. Child Fam. Stud..

[58] Luke A., Luck K.E., Doucet S. (2020). Experiences of Caregivers as Clients of a Patient Navigation Program for Children and Youth with Complex Care Needs: A Qualitative Descriptive Study. Int. J. Integr. Care.

[59] Palit A., Chatterjee A.K. (2006). Parent-to-Parent Counseling-a Gateway for Developing Positive Mental Health for the Parents of Children That Have Cerebral Palsy with Multiple Disabilities. Int. J. Rehabil. Res..

[60] Preyde M., Ardal F. (2003). Effectiveness of a Parent “Buddy” Program for Mothers of Very Preterm in a Neonatal Intensive Care Unit. Can. Med. Assoc. J..

[61] Ramos A., Cooke F., Miller E., Herbert L. (2021). The Food Allergy Parent Mentoring Program: A Pilot Intervention. J. Pediatr. Psychol..

[62] Shilling V., Bailey S., Logan S., Morris C. (2015). Peer Support for Parents of Disabled Children Part 1: Perceived Outcomes of a One-to-One Service, a Qualitative Study. Child Care Health Dev..

[63] Vadasy P.F., Meyer D.I., Fewell R.R., Greenberg M.T. (1985). Supporting Fathers of Handicapped Young Children: Preliminary Findings of Program Effects. Anal. Interv. Dev. Disabil..

[64] Winch A.E., Christoph J.M. (1988). Parent-to-Parent Links: Building Networks for Parents of Hospitalized Children. Child Health Care.

[65] Sullivan-Bolyai S., Bova C., Leung K., Trudeau A., Lee M., Gruppuso P. (2010). Social Support to Empower Parents (STEP): An Intervention for Parents of Young Children Newly Diagnosed with Type 1 Diabetes. Diabetes Educ..

[66] Dawson A. (1997). Parent-to-Parent Link Program. Can. J. Rehabil..

[67] Friedman Narr R., Kemmery M. (2015). The Nature of Parent Support Provided by Parent Mentors for Families with Deaf/Hard-of-Hearing Children: Voices from the Start. J. Deaf. Stud. Deaf. Educ..

[68] Lammers E.J., Zickafoose J.S., Peterson G.G., Blue L., Stewart K.A., Kranker K. (2019). Parent Partners: Evaluation of a Novel Peer-Support Intervention for the Caregivers of Children Hospitalized for Behavioral Health Conditions. Acad. Pediatr..

[69] Levasseur M.A., Ferrari M., McIlwaine S., Iyer S.N. (2019). Peer-Driven Family Support Services in the Context of First-Episode Psychosis: Participant Perceptions from a Canadian Early Intervention Programme. Early Interv. Psychiatry.

[70] Searcy S., Lee-Lawson C., Trombino B. (1995). Mentoring New Leadership Roles for Parents of Children with Disabilities. Remedial Spéc. Educ..

[71] Voos K.C., Miller L., Park N., Olsen S. (2015). Promoting Family-Centered Care in the NICU through a Parent-to-Parent Manager Position. Adv. Neonatal Care.

[72] Fratantoni K., Soghier L., Kritikos K., Jacangelo J., Herrera N., Tuchman L., Glass P., Streisand R., Jacobs M. (2022). Giving Parents Support: A Randomized Trial of Peer Support for Parents after NICU Discharge. J. Perinatol..

[73] Nelson K.A., Highstein G., Garbutt J., Trinkaus K., Smith S.R., Strunk R.C. (2012). Factors Associated with Attaining Coaching Goals during an Intervention to Improve Child Asthma Care. Contemp. Clin. Trials.

[74] Carter B., Flynn A., McKenna J. (2022). Parents Reaching Out to Parents: An Appreciative, Qualitative Evaluation of Stakeholder Experiences of the Parent Champions in the Community Project. Children.

[75] Iadarola S., Pellecchia M., Stahmer A., Lee H.S., Hauptman L., Hassrick E.M.G., Crabbe S., Vejnoska S., Morgan E., Nuske H. (2020). Mind the Gap: An Intervention to Support Caregivers with a New Autism Spectrum Disorder Diagnosis Is Feasible and Acceptable. Pilot Feasibility Stud..

[76] Jamison J., Baker N., Lopez M., Bearman S.K. (2023). An Analysis of Six Month Follow-Up Data from a Peer Parent Support Study. Adm. Policy Ment. Health Ment. Health Serv. Res..

[77] Liang W.H., Madan-Swain A., Cronin R.M., Jackson G.P. (2018). Development of a Technology-Supported, Lay Peer-to-Peer Family Engagement Consultation Service in a Pediatric Hospital. AMIA Annu. Symp. Proc..

[78] Dodds R.L. (2021). Meeting Families Where They Are: Text-Based Support in Parent to Parent Programs. Child Care Health Dev..

[79] Luelmo P., Kasari C. (2021). Randomized Pilot Study of a Special Education Advocacy Program for Latinx/Minority Parents of Children with Autism Spectrum Disorder. Autism.

[80] Kutash K., Duchnowski A.J., Green A.L., Ferron J.M. (2011). Supporting Parents Who Have Youth with Emotional Disturbances through a Parent-to-Parent Support Program: A Proof of Concept Study Using Random Assignment. Adm. Policy Ment. Health Ment. Health Serv. Res..

[81] DeNardo B.A., Stebulis J.A., Tucker L.B., Schaller J.G. (1995). Parents of Children with Rheumatic Disease as Peer Counselors. Arthritis Rheum..

[82] Garbutt J.M., Yan Y., Highstein G., Strunk R.C. (2015). A Cluster-Randomized Trial Shows Telephone Peer Coaching for Parents Reduces Children’s Asthma Morbidity. J. Allergy Clin. Immunol..

[83] Jarrett M.H. (1996). Parent Partners: A Parent-To-Parent Support Program in the NICU Part II: Program Implementation. Pediatr. Nurs..

[84] Reeves G.M., Wehring H.J., Connors K.M., Bussell K., Schiffman J., Medoff D.R., Tsuji T., Walker J., Brown A., Strobeck D. (2015). The Family Value of Information, Community Support, and Experience Study: Rationale, Design, and Methods of a “Family-Centered” Research Study. J. Nerv. Ment. Dis..

[85] Vohr B., McGowan E., Keszler L., Alksninis B., Hawes K., Tucker R. (2016). Impact of a Transition Home Program on Rehospitalization Rates of Preterm Infants. J. Pediatr..

[86] Berry-Carter K., Barnett B., Canavera K., Baker J.N., Mandrell B.N. (2021). Development of a Structured Peer Mentoring Program for Support of Parents and Caregivers of Children with Cancer. J. Pediatr. Nurs..

[87] Jensen L. (1999). Together Let’s Cope: Model for Parent Support Programs in the Neonatal Intensive Care Unit and Special Care Nursery. Mother Baby J..

[88] Wodinski L.M., Mattson McCrady H.M., Oswald C.M., Lyste N.J.M., Forbes K.L.L. (2017). Family Bedside Orientations: An Innovative Peer Support Model to Enhance a Culture of Family-Centred Care at the Stollery Children’s Hospital. Paediatr. Child Health.

[89] Dodds R.L., Walch T.J. (2022). The Glue That Keeps Everybody Together: Peer Support in Mothers of Young Children with Special Health Care Needs. Child Care Health Dev..

[90] Hilliard M.E., Tully C., Monaghan M., Wang J., Streisand R. (2017). Design and Development of a Stepped-Care Behavioral Intervention to Support Parents of Young Children Newly Diagnosed with Type 1 Diabetes. Contemp. Clin. Trials.

[91] Thomson G., Balaam M.C. (2019). International Insights into Peer Support in a Neonatal Context: A Mixed-Methods Study. PLoS ONE.

[92] Thomson G., Balaam M.C. (2021). Sharing and Modifying Stories in Neonatal Peer Support: An International Mixed-Methods Study. Scand. J. Caring Sci..

[93] Leggatt M.S. (2007). Minimising Collateral Damage: Family Peer Support and Other Strategies. Med. J. Aust..

[94] Wasilewski M.B., Kokorelias K.M., Nonoyama M., Dale C., McKim D.A., Road J., Leasa D., Tandon A., Goldstein R., Rose L. (2022). The Experience of Family Caregivers of Ventilator-Assisted Individuals Who Participated in a Pilot Web-Based Peer Support Program: A Qualitative Study. Digit. Health.

[95] Hornby G., Murray R., Jones R. (1987). Establishing a Parent to Parent Service. Child Care Health Dev..

[96] Smith A.V. (1986). Parent Outreach in a Neonatal Intensive Care Nursery. Soc. Work..

[97] Yingling M.E., Hock R.M., Feinberg M.E., Holbert A.A. (2020). Community-Engaged Process to Adapt Evidence-Based Programs for Parents of Children with Autism Spectrum Disorder. Child. Youth Serv. Rev..

[98] Dew A., Collings S., Dowse L., Meltzer A., Smith L. (2019). ‘I Don’t Feel like I’m in This on My Own’: Peer Support for Mothers of Children with Intellectual Disability and Challenging Behaviour. J. Intellect. Disabil..

[99] Liu K. (2018). A Parent-to-Parent Program in Taiwan. Infants Young Child.

[100] Mackey E.R., Herbert L., Monaghan M., Cogen F., Wang J., Streisand R. (2016). The Feasibility of a Pilot Intervention for Parents of Young Children Newly Diagnosed with Type 1 Diabetes. Clin. Pract. Pediatr. Psychol..

[101] Dahan S., Bourque C.J., Reichherzer M., Prince J., Mantha G., Savaria M., Janvier A. (2020). Peer Support Groups for Families in Neonatology: Why and How to Get Started?. Acta Paediatr. Int. J. Paediatr..

[102] Donegan A., Boyle B., Crandall W., Dotson J.L., Lemont C., Moon T., Kim S.C. (2016). Connecting Families: A Pediatric IBD Center’s Development and Implementation of a Volunteer Parent Mentor Program. Inflamm. Bowel Dis..

[103] Konrad S.C. (2007). What Parents of Seriously Ill Children Value: Parent-to-Parent Connection and Mentorship. Omega J. Death Dying.

[104] Nixon H.L. (1988). Reassessing Support Groups for Parents of Visually Impaired Children. J. Vis. Impair. Blind..

[105] Hall T.A., Mastel S., Nickel R., Wainer A. (2019). Parents Training Parents: Lessons Learned from a Study of Reciprocal Imitation Training in Young Children with Autism Spectrum Disorder. Autism.

[106] Stauber S.R., Mahan C.K. (1987). Successes and Struggles of Parent Support Groups in Neonatal Intensive Care Units. J. Perinatol..

[107] Garrand S., Sherman N., Rentchler D., Jung A.L. (1978). A Parent-to-Parent Program. Fam. Community Health.

[108] Santelli B., DiVenere N., Yoder J., De Carolis K. (2000). Supporting Parents: The Cornerstone of Parent to Parent. Except. Parent.

[109] Wong T.S.M., Shorey S. (2022). Experiences of Peer Support amongst Parents of Children with Neurodevelopmental Disorders: A Qualitative Systematic Review. J. Pediatr. Nurs..

[110] Flores G., Bridon C., Torres S., Perez R., Walter T., Brotanek J., Lin H., Tomany-Korman S. (2009). Improving Asthma Outcomes in Minority Children: A Randomized, Controlled Trial of Parent Mentors. Pediatrics.

[111] Koch K., Jones B. (2018). Supporting Parent Caregivers of Children with Life-Limiting Illness. Children.

