# Supplementary material for: Practical and Effective Mentorship Strategies for Caregivers of Children with Chronic Conditions: A Scoping Review
Source: Int J Environ Res Public Health. 2025 Feb 25;22(3):339. doi: 10.3390/ijerph22030339 (PMC11942062; doi:10.3390/ijerph22030339)
Supplement: Supplementary file 1 [file ijerph-22-00339-s001.zip › Table S1.pdf]

Table 1: MEDLINE (Ovid) Full search string used to identify available peer-reviewed literature.

Ovid MEDLINE(R) ALL <1946 to May 03, 2023>

Caregivers/
exp Parents/
exp parent-child relations/ or parenting/
maternal behavior/ or paternal behavior/
exp Legal Guardians/
(care giver* or caregiver* or carer* or dad or dads or father* or grandfather* or grandmother* or grandparent* or guardian* or maternal or mom or moms or mother* or parent* or paternal* or stepparent*).tw,kf.
or/1-6 [Caregiver]
Mentors/ or Mentoring/
Social Support/
Self-Help Groups/
Counseling/
(counsel* or coach* or guidance or mentor* or mentee* or navigat* or sponsor*).tw,kf.
self help group*.tw,kf.
or/8-13 [Mentorship and Self Help Groups]
exp peer group/
("peer direct*" or "peer led" or "peer run" or "peer lead").tw,kf.
carer* to carer*.tw,kf.
caregiver* to caregiver*.tw,kf.
care giver* to care giver*.tw,kf.
peer* to peer*.tw,kf.
caring for the caregiver*.tw,kf.
caring for caregiver*.tw,kf.
parent to parent.tw,kf.
(peer adj1 (coach* or counsel* or mediated* or mentee* or mentor* or support*)).tw,kf.
(parent* adj1 (mentee* or mentor*)).tw,kf.
self help group*.tw,kf.
or/15-26 [Peer to Peer]
7 and 14 and 27 [Peer-to-Peer Mentorship and Support for Caregivers]
exp Child/ or exp infant/ or adolescent/ or exp pediatrics/ or child, abandoned/ or exp child, exceptional/ or child, orphaned/ or child, unwanted/ or minors/ or (pediatric* or paediatric* or child* or newborn* or congenital* or infan* or baby or babies or neonat* or pre-term or preterm* or premature birth* or NICU or preschool* or pre-school* or kindergarten* or kindergarden* or elementary school* or nursery school* or (day care* not adult*) or schoolchild* or toddler* or boy or boys or girl* or middle school* or pubescen* or juvenile* or teen* or youth* or high school* or adolesc* or pre-pubesc* or prepubesc*).mp. or (child* or adolesc* or pediat* or paediat*).jn. [UAlberta Pediatric Filter]
28 and 29
